# Validation of a Sexual Function Survey for Transwomen After Vaginoplasty

**DOI:** 10.1097/og9.0000000000000135

**Published:** 2025-11-25

**Authors:** Rachel Pope, Amine Sahmoud, Alicia Castellanos, Erika Kelley, Stephen Rhodes, Grace Pelfrey, Jessica Abou Zeki, Kirtishri Mishra, Shubham Gupta

**Affiliations:** Urology Institute and Department of Obstetrics and Gynecology, University Hospitals Cleveland Medical Center, Cleveland, Ohio; and Case Western Reserve University School of Medicine, Cleveland, Ohio.

## Abstract

**OBJECTIVE:**

To describe the final validation processes for the final English-language tool to assess sexual function and satisfaction after gender-affirming vaginoplasty.

**METHODS:**

This was a quantitative and qualitative validation study. The 32-question SatisFunction survey was distributed to 50 individuals after vaginoplasty along with the Female Sexual Distress Scale for divergent validity testing. Thirty of these 50 participants then underwent one-on-one cognitive interviews with a member of the research team. The cognitive interviews assessed the construct validity of the survey questions based on the participants' responses. A Community Advisory Board and content expert team reviewed the results of the cognitive interviews to create a final version to be further tested. The revised survey was then distributed to 100 individuals for final validation.

**RESULTS:**

Cognitive interviews demonstrated 99.0% concordance between participants' survey responses and verbal confirmations, supporting interpretive reliability. Strong internal consistency was observed, with each domain significantly correlating with the total score (eg, Anatomy *r*=0.856, Arousal *r*=0.767, Orgasm *r*=0.748; all *P*<.001). Expected interdomain relationships were identified, including Arousal and Orgasm (*r*=0.552, *P*<.001). Female Sexual Distress Scale–Revised scores correlated negatively with Satisfaction (*r*=−0.416, *P*<.001), Desire (*r*=−0.302, *P*=.003), Genital Self-Image (*r*=−0.216, *P*=.034), and Total SatisFunction Score (*r*=−0.304, *P*=.003), supporting divergent validity. Factor analysis supported an eight-factor structure aligning with survey domains.

**CONCLUSION:**

This survey has now been developed and validated through a seven-phase process incorporating community input, physician/surgeon and psychologist expertise, and correlation to other surveys and can be reliably used clinically and in research.

## Abstract 2

This is a multiphased process of validating the first English-language survey for assessing sexual function after gender-affirming vaginoplasty.

Transgender and gender-diverse (TGD) individuals are individuals whose gender identity does not align with the sex and associated gender assigned to them at birth, contrasting with cisgender individuals, whose gender identity matches this assignment.^1,2^ Surgical outcomes indicate increased life satisfaction postoperatively, with high satisfaction rates reported among transfeminine individuals after vaginoplasty.^3^ Sexual function after vaginoplasty is a critical outcome for patients and surgeons alike. Objective measures are needed to guide patients on surgical techniques, risks, and outcomes. Unfortunately, currently available surveys fall short of addressing the sexual concerns of many of our patients after surgery. One survey, the Operated Male to Female Sexual Function Index, lacks aspects that patients expressed to us as being important in structured interviews such as specific anatomic assessments and is validated only in Italian and French.^4,5^ Therefore, our team decided to create and validate a survey using community-based research techniques over the course of seven phases. Our research team includes four gender-affirming surgeons (two urologists and two gynecologists with specialization in sexual medicine) and a psychologist who provides sexual therapy for patients undergoing vaginoplasty.

Phases 1–4 focused on content validation and have been reported previously.^6^ During phase 1, our team first reviewed the literature to identify the question types and surveys that included relevant questions in order to include time-tested questions and developed a 26-item draft survey.^7^ The survey was divided into eight domains and subjected to rigorous feedback and validation processes. Phase 2 involved the distribution of the survey to 16 TGD individuals who had undergone vaginoplasty at least 3 months prior, with subsequent cognitive interviews to gauge clarity, appropriateness of questions, and overall user experience. Cognitive interviewing in our study refers to engaging participants during the interview to share their thoughts aloud for each question of the survey, demonstrating their understanding of the question, and the appropriateness of the answer choices.^8^ Phase 3 involved further refinement through feedback from the five-person expert panel. This phase aimed at refining language, improving the comprehensibility of questions, and ensuring inclusivity across different aspects of sexual function and satisfaction to create the third survey draft. A Community Advisory Board was created, with five transgender women selected from the greater Cleveland, Ohio, area with diverse personal and surgical backgrounds. They reviewed the survey and ensured that it reflected diverse TGD individuals’ experiences and perspectives. The fourth draft of the survey expanded to 32 questions organized into eight domains: Genital Self Image, Desire, Arousal, Lubrication, Orgasm, Satisfaction, Pain, and Anatomy. Sahmoud et al^6^ describe the further adjustments made in phases 1–4.

Here, we discuss the final validation process during which construct, divergent, and internal validity were explored.^9^ Construct validity is the degree to which an assessment tool measures the theoretical construct it is intended to measure, in this case sexual function and satisfaction post vaginoplasty. Divergent validity is the ability of an assessment tool to distinguish between unrelated constructs. Internal validity refers to the degree to which the relationships among survey items and domains are consistent with the construct the instrument is intended to measure and assesses whether items group together as expected and whether the overall structure reflects the theoretical framework guiding survey development. The authors conclude that this validation process supports the clinical use of the survey for North American English speakers.

## METHODS

**Figure 1.**
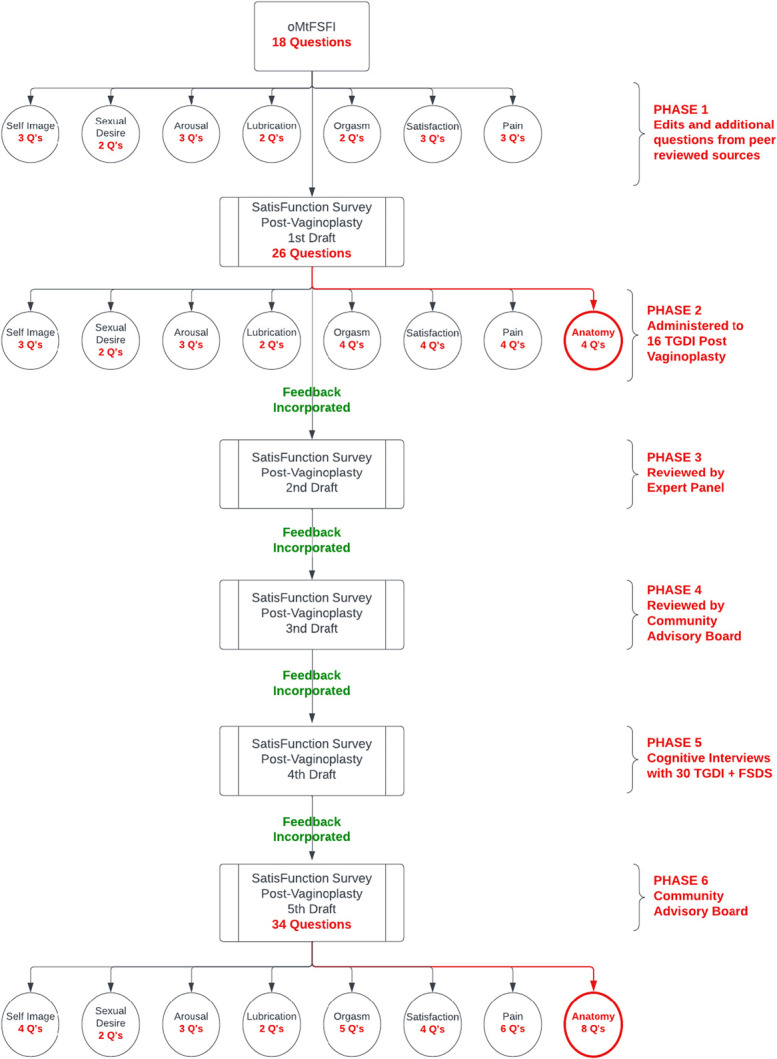
SatisFunction survey validation process. Sequential phases of survey development and validation phases 1–6, incorporating transgender and gender-diverse (TGD) individuals’ community input, expert review, cognitive interviewing, and iterative refinement to produce the final 34-item, eight-domain SatisFunction survey. oMtFSFI, Operated Male to Female Sexual Function Index; Q, question; FSDS, Female Sexual Distress Scale.

The University Hospitals Cleveland Medical Center IRB approved this study (STUDY20221324). For all seven phases of the study, participants were required to be 18 years of age or older and to have undergone vaginoplasty at least 3 months before survey administration to be included (Fig. 1). Participants were recruited through patient visits and compensated for their participation. In phase 5, the 32-question SatisFunction survey was distributed to 50 TGD individuals after vaginoplasty along with the Female Sexual Distress Scale for assessment of divergent validity. Of these 50 respondents, 30 underwent one-on-one cognitive interviews with a member of the research team. Four research team members functioned as interviewers. Cognitive interviews conducted 1 month after survey administration assessed temporal validity, test–retest reliability, and the construct validity of survey items. To assess test–retest reliability and temporal validity, interviewers reviewed each of the survey questions and asked participants to confirm or clarify their responses to assess concordance. During phase 6, the Community Advisory Board again reviewed the survey for final overall feedback over a Zoom meeting with research team members. This meeting included going through each of the 32 questions, determining any areas of overall concern in terms of language used, answer choices, and any missing aspects of sexual function or satisfaction. This meeting also involved analyzing the data from the 30 interviews and lasted 1 hour 30 minutes in total. The Community Advisory Board brought up multiple aspects to improve the survey that were incorporated and approved by the research team. The finalized 34-question survey was called the SatisFunction Survey Post Vaginoplasty (Appendix 1, available online at http://links.lww.com/AOG/E438). The revised survey was reviewed and finalized by the research team of gender-affirming care specialists and then distributed to 100 individuals for final validation.

*Pope. SatisFunction Survey After Trans Vaginoplasty. Obstet Gynecol 2025*.

Responses to SatisFunction questions were assigned a score of −2 to +2, with a more positive score indicating a more positive outcome (ie, greater satisfaction, less pain). Responses of “I don't know/prefer not to say” or “not applicable” were assigned a score of 0. The resulting question scores were then calculated within each domain and across domains to produce a total score. A detailed scoring guide, including domain-specific item mappings, canal-specific omissions, and scoring instructions, is provided in Appendix 2, http://links.lww.com/AOG/E438. SatisFunction subdomains were assessed for correlation to the Female Sexual Distress Scale through the Pearson correlation. Exploratory factor analysis was used to assess the clustering of responses and compared with the eight domains devised through the previously described process. The Horn parallel analysis was used to determine the number of factors to extract. Principal axes factor analysis with oblimin rotation was used to estimate factor loadings. Analysis was conducted with R 4.4.2 and used the psych package.^10,11^ The following describes the final validation process.

## RESULTS

One hundred individuals responded to the survey; however, four did not provide a vaginoplasty type or responded that they were unsure of type. These individuals were therefore excluded, leaving 96 responses for analysis. The median age was 35.5 years (range 20–72 years); 83.3% (n=80) were White, 13.5% (n=12) were Black, 3.1% (n=3) were American Indian, 1.0% (1) were unknown. Two individuals were Hispanic. Most had completed some college (n=28), college (n=34), or graduate school or more (n=18); 61.5% were employed (n=59). Nearly half (41.7%, n=40) lived on an income less than $30,000. Most (n=82) identified as transwoman, although a few identified as nonbinary (n=5) or other (n=9). Of those who answered, most were bisexual or pansexual (51.5%, n=52), 17.7% (n=17) were heterosexual, and 17.7% (n=17) were homosexual. In addition, 41.7% were single (n=40), nearly one-third were married or in a long-term committed relationship (n=28), and 18.8% (n=18) were in an open relationship. The median time on gender-affirming hormones was 6 years (range 1–50 years). Many had what they considered strong or very strong social support (36.5%, n=35). The Female Sexual Distress Scale was scored according to their published scoring criteria.

As previously discussed, phases 1–4 focused on clarification and comprehension for content validity, providing definitions of terms in the question stems, including more questions on specific anatomic locations, addressing genital self-image gender dysphoria, and specifying type of vaginoplasty, including only questions relevant to those with or without a vaginal canal.^6^ During phase 5 cognitive interviews, concordance was determined through accuracy in the participants' ability to confirm their comprehension of each question and their certainty in their responses. In totality, the 30 participants confirmed a 99.0% concordance rate for the survey overall between the construct each question was intended to assess and what participants said it addressed. Rare reasons for lack of concordance for participants included not having experienced an aspect of sexual activity, personal trauma, or postoperative healing complications. Participants also provided their reasoning for their survey answer choices during their interviews. Multiple participants discussed pain with sexual activity both alone and with partners, describing the pain more with arousal or initiation of sexual intimacy than any specific sexual act. Participants also reinforced omitting questions regarding vaginal canals if they did not have one.

During phase 6, the Community Advisory Board again provided feedback through a Zoom meeting. Each question was reassessed for language comprehension, and the survey overall was further examined for any gaps in assessing sexual function or satisfaction. Data and feedback from phase 5 cognitive interviews were also thoroughly discussed with the Community Advisory Board members. This meeting resulted in the inclusion of two new questions on pain and intensity with arousal as a result of both information gathered from participant comments and experiences of the Community Advisory Board. It was also highlighted during the meeting that the answer choices for each question included only the positive ends of the spectrum for each topic or indicated its absence (eg, very comfortable, moderately comfortable, slightly comfortable, not comfortable at all). The consensus was that without the inclusion of the negative answer choices, the survey does not allow the full spectrum of experiences to be captured. Thus, the answer choices for each question were changed to fit a different format (eg, very comfortable, slightly comfortable, neither comfortable or uncomfortable, slightly uncomfortable, very uncomfortable). In discussions with the Community Advisory Board, participants indicated that negative answer choices provide a wider array of options and an ability to improve the assessment of the patient experience. Scoring was then converted to a +2 to −2 scale based on Likert-type responses. Eight domains are covered by the 34-item survey (Appendix 1, http://links.lww.com/AOG/E438). Table 1 provides a brief description of them.

**Table 1.** Domains of the SatisFunction Survey

Domain	Focus
1. Genital self-image	Comfort with genital appearance when alone or with a partner
2. Desire	Interest in sexual activity, including desire, receptiveness to initiation, and sexual fantasies
3. Arousal	Mental and physical signs of sexual excitement such as genital warmth, tingling, or muscle contractions
4. Lubrication	Experience of internal (endogenous) vs external (exogenous) lubrication; includes reporting of unwanted secretions or discharge
5. Orgasm	Ability to orgasm and orgasm quality, with questions specific to clitoral, vaginal, or anal stimulation
6. Satisfaction	Overall satisfaction with sexual experiences since surgery
7. Pain	Experience and location of sexual pain
8. Anatomy	Perception of functional anatomy; includes targeted questions to assess postsurgical anatomic experience

Each domain represents a distinct aspect of sexual satisfaction and function assessed in the survey, reflecting both psychologic and physiologic experiences after vaginoplasty.

**Figure 2.**
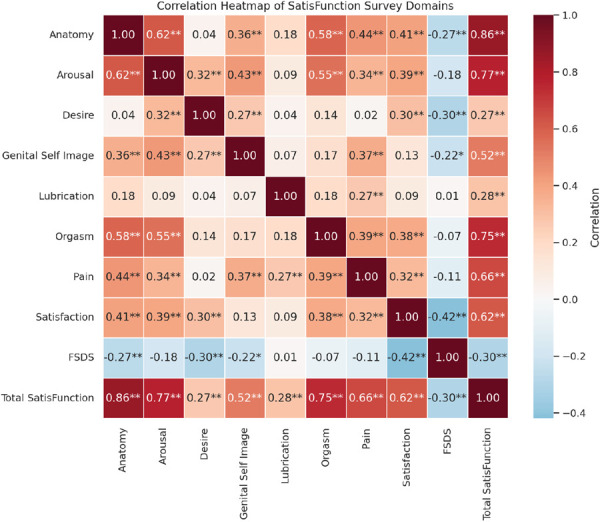
Phase 7 correlation heat map. This figure is a correlation heat map assessing correlation between the SatisFunction subdomains and between SatisFunction and the Female Sexual Distress Scale (FSDS) using the Pearson correlation. *P<.05, **P<.01, ***P<.001.

One hundred individuals completed the finalized survey, and 96 were included in the analysis. Correlations between each subdomain of SatisFunction were examined to assess the internal structure and validity of the finalized SatisFunction Survey (Fig. 2). Expected interdomain relationships were confirmed, including significant correlations between Arousal and Orgasm (*r*=0.552, *P*<.001) and Orgasm and Satisfaction (*r*=0.378, *P*<.001).

*Pope. SatisFunction Survey After Trans Vaginoplasty. Obstet Gynecol 2025*.

To evaluate divergent validity, we correlated domain scores and total scores with the Female Sexual Distress Scale. As hypothesized, higher SatisFunction scores correlated negatively with Female Sexual Distress Scale scores, with notable correlations in domains such as Satisfaction (*r*=−0.416, *P*<.001), Desire (*r*=−0.302, *P*=.003), Genital Self-Image (*r*=−0.216, *P*=.034), and Anatomy (*r*=−0.266, *P*=.009). No significant correlations were observed between Female Sexual Distress Scale scores and physiologic domains such as Lubrication, Orgasm, and Pain. These findings support the internal consistency, construct validity, and divergent validity of the SatisFunction Survey.

**Figure 3.**
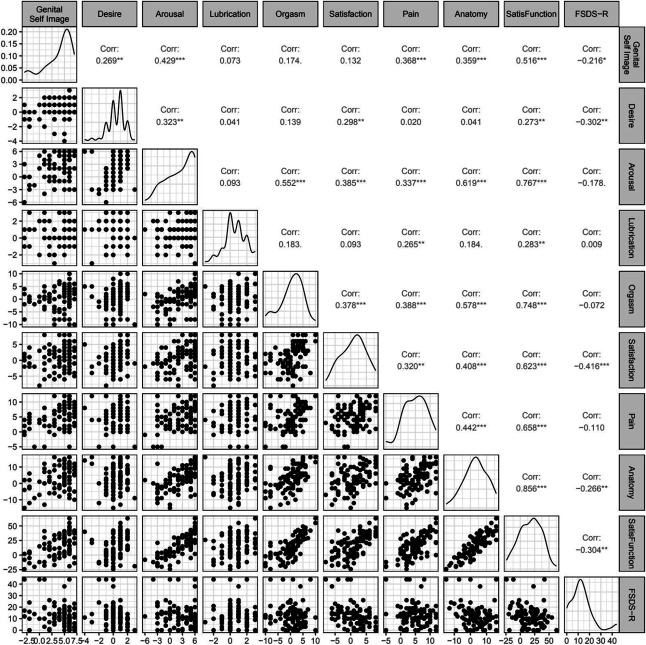
Matrix of bivariate scatterplots displaying the relationships among SatisFunction domain scores, total SatisFunction score, and the Female Sexual Distress Scale–Revised (FSDS-R). Each plot visualizes the distribution and linear association between two measures. Strong positive correlations (corrs) between domains demonstrate internal consistency; negative correlations between FSDS-R and key domains support divergent validity. *P<.05, **P<.01, ***P<.001.

Figure 3 presents scatterplots of the bivariate relationships between scores on the different measures used. SatisFunction negatively correlates with sexual dysfunction as measured by the Female Sexual Distress Scale–Revised (*r*=−0.31, 95% CI, −0.48 to −0.12, *P*<.01). Exploratory factor analysis was conducted to assess how clustering of different items compares with the eight domains devised through the development process outlined previously. The Horn parallel analysis suggested extraction of five factors; Table 2 presents the factor loadings for each question in SatisFunction. We assessed the themes of the questions loading highly (absolute loading more than 0.3) on a given factor. Factor 1 relates primarily to questions covering general genital sensation. Factor 2 relates to the questions covering arousal/desire. Factor 3 included questions on orgasm and sexual sensation. For factor 4, the strongest loadings were for questions on pain. Finally, factor 5 relates to aspects of anatomy (circumference, depth). All of these factors proved to be important for patients when discussing responses through the cognitive interviews.

*Pope. SatisFunction Survey After Trans Vaginoplasty. Obstet Gynecol 2025*.

**Table 2.** Factor Loadings From Exploratory Factor Analysis of SatisFunction Survey Items (N=96)

SatisFunction Survey Question	PA2	PA1	PA3	PA4	PA5
Anatomy question 5: How would you rate the sensitivity of your labia minora (inner lips of the vulva) (sensitivity describes awareness of light touch)?	0.834	0.083	−0.008	0.004	−0.063
Anatomy question 3: How would you rate the sensitivity of your labia majora (outer lips of the vulva) (sensitivity describes awareness of light touch)?	0.786	0.037	−0.028	−0.079	−0.019
Anatomy question 6: How satisfied are you with the sensitivity of your labia minora (inner lips of vulva) (sensitivity describes awareness of light touch)?	0.758	0.196	−0.036	0.113	−0.014
Anatomy question 4: How satisfied are you with the sensitivity of your labia majora (outer lips of vulva) (sensitivity describes awareness of light touch)?	0.725	0.193	−0.065	0.073	0.081
Pain question 1: How often do you experience pain during vaginal penetration?	0.589	−0.324	0.016	0.219	0.348
Orgasm question 3: How often have you been able to achieve an orgasm with vaginal penetration?	0.513	−0.146	0.398	−0.078	0.191
Satisfaction question 2: How satisfied are you with the feeling of vaginal penetration?	0.494	0.011	0.25	0.061	0.328
Pain question 2: How would you rate the intensity of pain you experience during vaginal penetration?	0.483	−0.308	−0.053	0.303	0.249
Orgasm question 5: How often have you been able to achieve an orgasm with anal penetration?	0.297	−0.016	0.287	0.055	0.24
Arousal question 1: How satisfied are you with the frequency of your sexual arousal, the mental and physical feelings of sexual excitement warmth, or tingling in the genitals/muscle contractions?	0.045	0.848	0.112	−0.001	0.09
Desire question 2: How satisfied are you with your level of sexual desire?	−0.029	0.761	−0.071	0.078	0.187
Arousal question 3: How satisfied are you with the intensity of your sexual arousal?	0.135	0.688	0.25	0.027	−0.015
Arousal question 2: How would you rate the intensity of your sexual arousal?	0.302	0.502	0.266	0.159	−0.059
Genital self-image question 2: How comfortable do you or would you feel with your partner(s) seeing your external genitalia?	0.336	0.446	−0.33	0.161	−0.156
Satisfaction question 1: How satisfied are you with the amount of sexual activity you have?	0.122	0.433	0.075	0.02	0.32
Desire question 1: How often do you feel sexual desire, have the feeling of wanting a sexual experience, feel receptive to a partner’s sexual initiation, or fantasize about having sex?	−0.226	−0.355	−0.011	−0.162	0.15
Genital self-image question 3: How often does scarring interfere with how comfortable you are with the physical appearance of your external genitalia?	0.342	0.346	−0.315	0.197	−0.14
Orgasm question 1: How often have you been able to have an orgasm when you wanted to?	−0.127	0.161	0.685	0.201	0.051
Orgasm question 4: How often have you been able to achieve an orgasm with clitoral stimulation?	−0.167	0.195	0.643	0.313	−0.013
Anatomy question 7: During vaginal penetration, how would you rate the sensitivity of your prostate erectile tissue between the bladder and rectum (sensitivity describes awareness of touch pressure)?	0.463	−0.088	0.545	−0.047	−0.101
Anatomy question 1: How would you rate the sensitivity of your clitoris (sensitivity describes awareness of light touch)?	0.228	0.278	0.514	−0.009	−0.167
Anatomy question 8: During vaginal penetration, how satisfied are you with the sensitivity of your prostate erectile tissue between bladder and rectum (sensitivity describes awareness of touch pressure)?	0.475	0.064	0.477	−0.133	−0.025
Orgasm question 2: How satisfied do you feel with the quality of your orgasm during sexual stimulation or intercourse?	−0.093	0.39	0.451	0.176	0.053
Anatomy question 2: How satisfied are you with the sensitivity of your clitoris (sensitivity describes awareness of light touch)?	0.266	0.282	0.407	0.181	−0.079
Pain question 6: How would you rate the intensity of pain you experience with arousal?	−0.045	−0.106	0.095	0.727	0.023
Pain question 3: How often do you experience pain with clitoral stimulation?	0.047	0.075	0.118	0.723	−0.12
Pain question 4: How would you rate the intensity of pain you experience with clitoral stimulation?	0.068	0.18	−0.088	0.654	−0.075
Pain question 5: How often do you experience pain with arousal?	−0.049	−0.13	−0.003	0.623	0.027
Lubrication question 2: How often do you feel unwanted secretions outside of sexual activity or intercourse?	−0.128	−0.068	−0.156	0.3	0.254
Genital self-image question 1: How comfortable are you with the physical appearance of your external genitalia?	0.14	0.023	0.023	0.27	0.096
Genital self-image question 4: Compared with before your procedure, rate your gender dysphoria in relation to your genitalia (gender dysphoria means discomfort or stress related to gender).	−0.206	0.226	−0.152	0.234	0.024
Satisfaction question 4: How satisfied are you with the circumference of your vagina?	0.025	0.191	−0.125	−0.086	0.78
Satisfaction question 3: How satisfied are you with the depth of your vagina?	−0.032	0.099	0.09	−0.035	0.712
Lubrication question 1: How often do you feel secretions during sexual activity or intercourse without using lubricants while engaging in sexual activity alone or with a partner?	0.227	0.037	0.218	−0.016	−0.228

This table presents factor loadings from a principal axis factor analysis with oblimin rotation, following the Horn parallel analysis to retain five factors. Bolded loadings represent the primary factor association for each item. PA1–PA5 represent the extracted factors, which correspond broadly to domains including Arousal/Desire, Orgasm/Anatomy, Pain, Genital Self-Image, and Satisfaction with Vaginal Canal Characteristics.

## DISCUSSION

Overall, the eight domains as developed through codesign with community and medical stakeholders suggest comprehensive sexual function assessment. This survey has now been validated through a seven-phase process incorporating community input, physician/surgeon and psychologist expertise, and correlation to other surveys. There is high internal consistency, and participants were able to comprehend the questions easily and felt that their responses reflected their true state. No significant correlations were observed between Female Sexual Distress Scale scores and physiologic domains such as Lubrication, Orgasm, and Pain, suggesting that distress may be more closely linked to psychologic and body image domains rather than specific physiologic responses. This could be further explored in additional research.

Strengths of this study include community participation from the TGD population, which is integral to the development of the survey through incorporation of key stakeholder feedback. The addition of a domain with questions targeting specific anatomy also strengthens the assessment of sexual function and satisfaction for this population. Weaknesses of the study include lack of testing over specific time points (6 months after surgery, 1 year after surgery, etc). We expected changes of functionality over time from surgery and therefore did not want surgical outcomes to directly affect the validation process. We made an effort to avoid inclusion of participants who might have experiences influenced by early postoperative changes; however, because everyone has potentially different postoperative courses, we cannot ensure that there is no effect.

Our sample of respondents could also be skewed toward positive or negative outcomes given motivational factors for completing research surveys; however, we found a reasonable balance represented in our sample. Time since vaginoplasty was not assessed or controlled for in this analysis, representing a limitation because postoperative sexual function and satisfaction may vary according to healing stage and time-dependent anatomic or psychologic changes.

We foresee wide distribution and use of the survey by national and international institutions treating TGD individuals after vaginoplasty. Although the survey has clear use in future research studies, we also envision its clinical use postoperatively at multiple time points (ie, 3 months, 6 months, 1 year) to compare surgical techniques and to assess outcomes after surgical revisions. We hope that this tool will assist clinicians in continuously identifying areas of improvement and objectively measuring outcomes of medical and surgical interventions. The tool also should help sexual medicine experts to evaluate specific domains of sexual function while still being feasible to use by nonexperts. It is important to include as comprehensive and as community-informed a measure as possible for future clinical and research use.
